# Early loss of glutathione ‐s‐ transferase (GST) activity during diverse forms of acute renal tubular injury

**DOI:** 10.14814/phy2.15352

**Published:** 2022-06-24

**Authors:** Richard A. Zager, Ali C. M. Johnson

**Affiliations:** ^1^ Fred Hutchinson Cancer Center Seattle Washington USA

## Abstract

Glutathione‐S‐transferases (GSTs) are a diverse group of phase II detoxification enzymes which primarily evoke tissue protection via glutathione conjugation to xenobiotics and reactive oxygen species. Given their cytoprotective properties, potential changes in GST expression during AKI has pathophysiologic relevance. Hence, we evaluated total GST activity, and the mRNA responses of nine cytosolic GST isotypes (GST alpha1, kappa1, mu1/5, omega1, pi1 sigma1, theta1, zeta1 mRNAs), in five diverse mouse models of AKI (glycerol, ischemia/reperfusion; maleate, cisplatin, endotoxemia). Excepting endotoxemia, each AKI model significantly reduced GST activity (~35%) during both the AKI “initiation” (0‐4 h) and “maintenance” phases (18 or 72 h). During the AKI maintenance phase, increases in multiple GST mRNAs were observed. However, no improvement in GST activity resulted. Increased urinary GST excretion followed AKI induction. However, this could not explain the reduced renal GST activity given that it also fell in response to ex vivo renal ischemia (i.e., absent urinary excretion). GST alpha, a dominant proximal tubule GST isotype, manifested 5–10‐fold protein increases following AKI, arguing against GST proteolysis as the reason for the GST activity declines. Free fatty acids (FFAs) and lysophospholipids, which markedly accumulate during AKI, are known to bind to, and suppress, GST activity. Supporting this concept, arachidonic acid addition to renal cortical protein extracts caused rapid GST activity reductions. Based on these results, we conclude that diverse forms of AKI significantly reduce GST activity. This occurs despite increased GST transcription/translation and independent of urinary GST excretion. Injury‐induced generation of endogenous GST inhibitors, such as FFAs, appears to be a dominant cause.

## Graphical abstract

The paper demonstrates that an important 'self defense' group of anti‐oxidant enzymes, the glutathione‐S‐transferases (GSTs), have suppressed activity following diverse forms of AKI. Data are presented that this is due to enzyme inhibition which is induced by free fatty acids which markedly accumulate during AKI due to phospholipid hydrolysis and decreased mitochondrial consumption. Thus, this paper provides new insights into AKI pathogenesis.

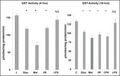

## 1 INTRODUCTION

Glutathione ‐S‐ transferases (GSTs) are a highly diverse group of phase II detoxification enzymes (Alkinson & Babbitt, 2009; Josephy, 2010; Knight et al., 2007; Rushmore & Pickett, 1993; Townsend & Tew, 2003). Their capacity to confer tissue protection stems, in large part, from their ability to conjugate glutathione to a wide variety of endogenous/exogenous electrophiles and reactive oxygen species, thereby mitigating their toxicities (Josephy, 2010; Knight et al., 2007; Rushmore & Pickett, 1993; Townsend & Tew, 2003). That GSTs confer a survival advantage likely explains their ubiquitous presence in virtually all life forms, and their up‐regulation in select pathologic states. As one example, GSTs are overly expressed in diverse forms of cancers, thereby increasing resistance to chemotherapeutic attack (e.g., ref. (Kennedy et al., 2020; Townsend & Tew, 2003).

GSTs are subtyped into cytosolic and membrane‐bound moieties. At least eight cytosolic subtypes have been identified, denoted by the Greek letters alpha, kappa, mu, omega, pi, sigma, theta, and zeta (e.g., ref. (Knight et al., 2007; Mar et al., 2015). Additionally, subtypes within each group exist which can be differentially expressed in different tissues and cell types (Knight et al., 2007; Mar et al., 2015).. Within kidney, the alpha and pi isotypes are predominantly located in proximal and distal tubules, respectively (Branten et al., 2000; Cummings et al., 2000; Knight et al., 2007; McMahon et al., 2019; Mohana & Achary, 2017; Walshe et al., 2009). Because of this, differential increases in urinary GST isotype excretion during AKI may serve as specific markers of tubular injury sites (Branten et al., 2000; Cummings et al., 2000; McMahon et al., 2019; Mohana & Achary, 2017; Walshe et al., 2009).

The fate of renal GST activity, and specific cytosolic GST gene expression, during AKI remain poorly defined. Hence, the present study has sought to determine the impact of diverse forms of AKI (ischemia‐reperfusion injury, myoglobin, maleate, cisplatin nephrotoxicity, endotoxemia) on both total renal cortical GST enzymatic activity and cytosolic GST isotype gene expression. Given that oxidative stress is an inducer of GSTs and occurs during diverse forms of AKI, its impact on specific GST isotype gene expression and activity in kidney was assessed.

## 2 METHODS

### 2.1 General information

All experiments were conducted using male CD‐1 mice (30–40 gms; obtained from Charles River Laboratories, Wilmington, MA). The protocols received IACUC approval from the Fred Hutchinson Cancer Center, Seattle, WA. At the completion of the protocols listed below, the mice were deeply anesthetized with pentobarbital (40–50 mg/Kg), the abdominal cavities were opened through a midline incision, a blood sample was collected from the vena cava for blood urea nitrogen (BUN) and plasma creatinine measurement, and then the kidneys were resected and iced. Following renal resection, renal cortical tissues were isolated and extracted for total RNA (RNeasy; Qiagen: Beverley, MA) and protein content (Pierce BCA Protein assay; PI23227). The mice had free food and water access throughout.

### 2.2 Analyses

BUN and creatinine concentrations were measured using commercially available kits (kits: Z50300168; Z5030020, respectively; Biochain, Newark, CA). GST isotype mRNAs. (alpha1, kappa1, mu1 mu5, omega1, pi1, sigma1, theta1, zeta1); were measured by competitive RT‐PCR, with the results factored by simultaneously obtained GAPDH product. The primer pairs used are presented in Table 1. Total GST enzymatic activity was measured in renal cortical protein extracts using a kit from Sigma Aldrich (CS0410). This assay utilizes 1‐chloro‐2,4‐dinitrobenzene (CDNB) as a substrate for GST‐ mediated glutathione (GSH) conjugation (exogenous GSH provided). Upon CDNB‐GSH conjugation, the product is quantified spectrophotometrically at 340 nm. The results were expressed as μmol conjugate/ mg issue protein/ min of incubation. Given that GST alpha 1 is thought to be a dominant GST isotype in proximal tubules as noted above, its protein concentration was measured in renal protein extracts using a GST alpha1‐ mouse specific‐ ELISA (Abcam; Boston, MA; ab283967). Results were expressed as ng/mg tissue protein.

**TABLE 1:** Mouse primers used for RT‐PCR

mRNA	Primer sequences	Product size
GST alpha1	5′‐ GACCAGAGCCATTCTCAACTA −3′ 5′‐ GTCAGAAGGCTGGCATCAA −3′	377 bp
GST kappa1	5′‐ GGAGAAGGTGTCCAGAGAGATA −3′ 5′‐ AGCGGTCAGACCCAAATAAC ‐3′	266 bp
GST mu1	5′‐ CACCAGCACCATGCCTATGA −3′ 5′‐ CTCCATCCAGGTGGTGCTTT −3′	275 bp
GST mu5	5′‐ CCCACAGCGTCCAGTATAAA −3′ 5′‐ GTATCTCAGGATGGCGTTACTC ‐3′	320 bp
GST omega1	5′‐ GCCCGAGTGGTTCTTTGAGA −3′ 5′‐ AACGCTTCCCTTAGGTTCGG −3′	261 bp
GST pi1	5′‐ TCAAGCCCACTTGTCTGTATG −3′ 5′‐ GGCCTTCACGTAGTCATTCTT −3′	236 bp
GST sigma1	5′‐ AGACAGCGTTGGAGCAATGT −3′ 5′‐ TGTGGTGCTGCAGATATCCC ‐3′	239 bp
GST theta1	5’‐ GATAGTCTGGCCAGTTACAGAAG −3′ 5′‐ CCAGTGGTCAGGAACCTTATAC ‐3′	295 bp
GST zeta1	5′‐ AGTGCCCATCAACCTCATAAA −3′ 5′‐ AGCGTTAAAGCCGGAAGTAATA −3′	325 bp
GAPDH	5′‐CTGCCATTTGCAGTGGCAAAGTGG‐3′ 5′‐TTGTCATGGATGACCTTGGCCAGG‐3′	437 bp

*Note*: Primers used for detection of renal cortical glutathione ‐S‐ transferase mRNA isotypes under control conditions and following acute renal injury.

### 2.3 Glycerol induced AKI

Ten mice were lightly anesthetized with isoflurane and injected intramuscularly with 50% glycerol in a dose of 8 mg/Kg, administered in equally divided doses into the hind limbs, as previously described (Zager et al., 2016). This model induces rhabdomyolysis, renal hypoperfusion, myohemoglobinuric cast formation, and heme Fe mediated oxidative stress (Zager, 1966). At either 4 or 18 h post glycerol injection (n,5 at each time point), the mice were sacrificed for blood and renal cortical tissue extraction with the samples being analyzed as noted above. The results were compared to control values, obtained from five normal mice.

### 2.4 Maleate induced nephrotoxicity

When injected into rodents, maleate undergoes proximal tubule cell uptake via organic anion transporters (OATs) (Zager et al., 2008). Following uptake, maleate causes severe coenzyme A depletion, thereby inhibiting mitochondrial activity which culminates in severe ATP depletion and oxidative stress (Zager et al., 2008). Hence, maleate represents a nephrotoxin that recapitulates key mediators of ischemia–reperfusion AKI (Zager et al., 2008). To assess whether maleate also reduces GST activity, 10 mice were injected intraperitoneally (IP) with 600 mg/Kg Na maleate. At either 4 or 18 h post injection, half of the mice were sacrificed to obtain plasma and renal cortical RNA and protein samples which were analyzed as noted above. The results were contrasted with those observed in five control mice.

### 2.5 Ischemia–reperfusion (I/R) induced AKI

Eight mice were deeply anesthetized with pentobarbital (40–50 mg/Kg), followed by bilateral renal pedicle occlusion × 25 min performed through a midline abdominal incision. Either 4 h or 18 h later (n,4 at each time point), plasma and renal tissues were obtained and analyzed as noted above. The results were compared to those observed in six sham‐operated mice (n,3 each at 4 and 18 h time points).

### 2.6 Cisplatin‐induced AKI

Six mice were subjected to intraperitoneal cisplatin injection (15 mg/Kg; in 500 μl saline). At 72 h they were deeply anesthetized with pentobarbital. Plasma and kidney samples were obtained through a midline abdominal incision for subsequent analyses as noted above.

### 2.7 Endotoxemia‐induced AKI

Unlike the four AKI models described above which induce marked proximal tubule necrosis/apoptosis and cast formation, endotoxemic‐induced AKI is predominantly mediated via hemodynamic changes (Wang et al., 2008; Zager et al., 2006). Thus, it represents a model that can be contrasted to those which have severe proximal tubule damage. To this end, 10 mice received 2 mg/Kg endotoxin (lipopolysaccharide, *E. coli* 0111:B4 in ∼800 μl of saline; L‐2630, Sigma). Plasma and renal tissues were obtained 4 or 18 h later and analyzed as noted above.

### 2.8 Mechanisms for GST depletion in response to AKI

Possible mechanisms for GST activity reductions during AKI include the following: (i) Decreased GST transcription (e.g., as assessed by mRNA measurements); (ii) increased GST urinary excretion; (iii) GST plasma efflux; (iv) increased GST proteolysis; or (v) accumulation of GST inhibitors. GST transcription was assessed by GST isoform mRNA responses to injury, as noted above. The remaining possibilities were considered in the experiments described below.

### 2.9 Urine GST excretion

To gauge urinary GST excretion, urine samples were collected from the bladders of five maleate treated mice at 4 h post maleate injection. Five normal mice provided control urine samples. Urinary GST activity was measured by the enzymatic assay as noted above. Values were expressed as μmol/mg creatinine/minute. The maleate model was chosen for this assessment as it manifested the greatest degree of renal cortical GST activity losses (see Results).

### 2.10 Ex vivo GST depletion

Given that GSTs could be lost from the kidney either into the circulation or into the urine, we sought to determine whether GST depletion can develop during ex vivo injury where neither blood flow nor urine flow are at issue. To this end, kidneys were extracted from 3 normal mice, and they were immediately incubated in PBS at either 4°C or 37°C PTS × 4 h. Cortical kidney samples were taken at baseline and after 2 and 4 h of incubation and assayed for GST activity, factored by total protein concentrations. The % changes in GST activity from baseline to 2 h and 4 h of incubation were assessed. Results obtained with 4°C versus 37°C incubations were also compared.

### 2.11 Impact of arachidonic acid on GST activity

A number of endogenous molecules, most notably free fatty acids (FFAs) and lysophospholipids (LPLs), bind to GSTs and inhibit their activity (e.g., ref. (Boyer et al., 1982; Dixon & Edwards, 2009; Mitra et al., 1991). Because AKI dramatically increases renal cortical FFA/LPL concentrations in kidney due to phospholipid hydrolysis (Venkatachalam et al., 1988; Zager et al., 1991; Zager et al., 1993), we tested whether arachidonic acid (C20:4) addition to a renal cortical protein extract would produce this result. To this end, normal kidney cortex was homogenized in a triton x‐100 based lysis buffer, pH 7.2, in the presence of protease inhibitors (Roche; 1,697,498). Each sample was centrifuged at 4°C × 12 g and the supernatants were saved for analysis. Each sample (*n* = 4) was divided into three aliquots (50 μM or 200 μM C20:4 final concentrations, or vehicle). Each aliquot was then assayed for GST enzymatic activity as noted above.

### 2.12 Impact of oxidant stress on renal GST isotype mRNAs, renal GST activity, and GST alpha protein concentrations

Because GST expression is impacted by oxidative stress, and because the latter is a mediator of diverse forms of AKI, the impact of a pro‐oxidant challenge on GST isotype gene induction was assessed. Ten mice were subjected to a tail vein injection of a pro‐oxidant challenge (I mg iron sucrose +1 μmole tin protoporphyriin; “RBT1”, from Renibus Therapeutics, Southlake, TX) as previously described (Johnson et al., 2017; Johnson & Zager, 2018; Zager et al., 2016). Ten saline vehicle‐injected mice served as controls. At either 4 or 18 h post injection, half of the RBT‐1 treated mice and control mice were anesthetized and the kidneys were removed and processed for total RNA and total protein, as noted above. All RNA samples were probed for each of the GST isotype mRNAs noted above. The 18 h protein samples were also assayed for GST enzymatic activity. GST alpha protein levels were also assessed. BUNs and plasma creatinine levels were also measured.

### 2.13 Calculations and statistics

All values are given as means ± 1 SEM. Statistical comparisons between two groups were made using unpaired Student's *t*‐test. The 95% confidence ranges for individual GST mRNAs and GST activity levels were calculated. Significance for individual sets of isotype mRNAs or GST activities within the AKI groups was assessed by whether its mean value fell outside of the 95% confidence ranges of control mice (i.e., *p* < 0.05).

## 3 RESULTS

### 3.1 GST activity in response to AKI

**FIGURE 1:**
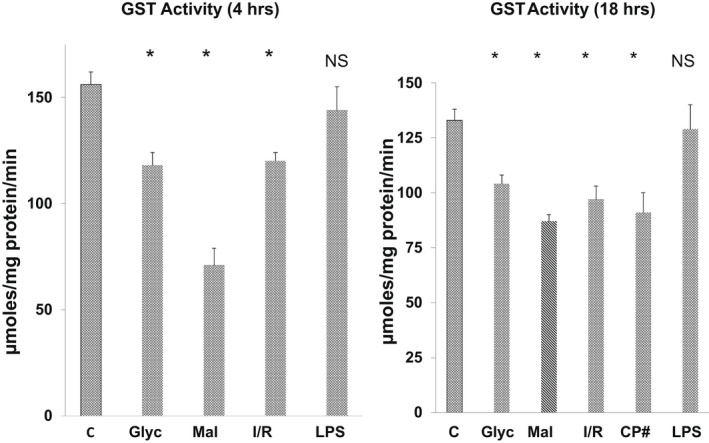
Legend. GST enzymatic activity following AKI induction. At both 4 and 18 h post glycerol (glyc), maleate (mal), and ischemic–reperfusion I/R) injury, significant decreases in GST activity were observed (* below the 95% confidence range for normal kidney protein extracts). Values are μmol of GST product/mg tissue protein/minute incubation (see text). Cisplatin (CP) associated GST activity levels were only assessed at 72 h post drug administration and revealed significant reductions.

As shown in Figure 1, there were significant reductions in total GST activity (μmol conjugate/mg issue protein/ min of incubation) within 4 h post glycerol, I/R, and maleate‐induced AKI. These reductions persisted unabated into the maintenance AKI phases. Comparable GST activity reductions were observed at 72 h post cisplatin administration (the only cisplatin time point assessed). In striking contrast to these results, LPS did not reduce GST activity at either 4 or 18 h post LPS administration. Absolute GST activity values are presented in Tables 2 and 3.

**TABLE 2:** Assessments performed at 4 h post AKI induction

mRNA	Control	Glycerol	Maleate	Ischemia	Cisplatin	LPS
GST alpha1	0.55 ± 0.07	0.84 ± 0.03	0.64 ± 0.08	0.78 ± 0.05	ND	0.37 ± 0.05
GST kappa1	0.67 ± 0.02	0.47 ± 0.08	0.59 ± 0.12	0.69 ± 0.09	ND	0.45 ± 0.02
GST mu1	0.44 ± 0.05	0.58 ± 0.09	0.40 ± 0.06	0.3 ± 0.05	ND	0.60 ± 0.16
GST mu5	0.68 ± 0.01	0.63 ± 0.02	0.64 ± 0.02	0.93 ± 0.07	ND	0.52 ± 0.02
GST omega1	0.22 ± 0.01	0.21 ± 0.02	0.44 ± 0.10	0.36 ± 0.09	ND	0.13 ± 0.02
GST pi1	0.32 ± 0.03	0.34 ± 0.03	0.27 ± 0.02	0.33 ± 0.02	ND	027 ± 0.06
GST sigma1	0.10 ± 0.02	0.13 ± 0.06	0.14 ± 0.02	0.18 ± 0.01	ND	0.11 ± 0.027
GST theta 1	0.54 ± 0.03	0.43 ± 0.03	0.71 ± 0.14	0.51 ± 0.14	ND	0.41 ± 0.2
GST zeta1	1.14 ± 0.05	1.24 ± 0.08	0.93 ± 0.06	1.79 ± 0.03	ND	0.98 ± 0.08
GST activity	156 ± 6	118 ± 6*	71 ± 8*	125 ± 4*	ND	144 ± 11
BUN	26 ± 2	57 ± 2*	52 ± 10*	69 ± 15*	ND	26 ± 4
Creatinine	0.35 ± 0.02	0.45 ± 0.2*	0.9 ± 0.16*	0.7 ± 0.04*	ND	0.30 ± 0.0

*Note*: GST mRNA isotypes, GST enzymatic activity, and BUN/creatinine concentrations under control conditions and 4 h following induction of the AKI protocols. Cisplatin assessments were not done (ND) at this time point. Excepting endotoxemia (LPS), GST activity was significantly depressed in all injury models (*; <0.05, i.e., below the control values 95% confidence range). However, no compensatory increases in GST mRNAs were observed. BUN and creatinine levels (mg/dl) were increased in all injury models, excepting endotoxemia.

**TABLE 3:** Assessments performed at 18 or 72 h post AKI induction

mRNA	Control	Glycerol	Maleate	Ischemia	Cisplatin*	LPS
GST alpha1	0.55 ± 0.07	4.5 ± 0.7*	5.4 ± 1.5*	2.3 ± 0.6*	2.3 ± 0.66 *	0.95 ± 0.18*
GST kappa1	0.67 ± 0.02	0.16 ± 0.01	0.29 ± 0.02	0.74 ± 0.04	0.38 ± 0.07	0.49 ± 0.05
GST mu1	0.44 ± 0.06	0.53 ± 0.07	0.75 ± 0.04	0.53 ± 0.05	0.76 ± 0.2*	0.8 ± 0.05
GST mu5	0.68 ± 0.01	0.64 ± 0.02	0.67 ± 0.08	0.89 ± 0.07	1.25 ± 0.02*	0.88 ± 0.16
GST omega1	0.22 ± 0.01	0.75 ± 0.14*	0.90 ± 0.10*	0.57 ± 0.03*	0.52 ± 0.15*	0.23 ± 0.02
GST pi1	0.32 ± 0.03	0.42 ± 0.05	0.72 ± 0.07*	0.44 ± 0.03	0.37 ± 0.02	0.28 ± 0.04
GST sigma1	0.07 ± 0.02	4.66 ± 1.04*	2.7 ± 0.48*	0.9 ± 0.13*	6.51 ± 2.1*	0.21 ± 0.10*
GST theta 1	0.54 ± 0.03	1.27 ± 0.05*	1.75 ± 0.19*	3.0 ± 0.96*	1.55 ± 0.22*	2.1 ± 0.63*
GST zeta1	1.14 ± 0.05	0.03 ± 0.05	0.64 ± 0.09	1.41 ± 0.12	0.72 ± 0.27	1.05 ± 0.02
GST activity	133 ± 4	104 ± 4*	87 ± 3*	97 ± 6*	91 ± 7*	129 ± 3.6
GSTa1 Protein	7.3 ± 0.3	26.0 ± 4.8*	15.9 ± 1.0*	15.6 ± 2.8*	18.1 ± 2.0*	9.4 ± 1.1
BUN	26 ± 2	164 ± 9*	138 ± 8*	122 ± 7*	137 ± 3*	76 ± 10*
Creatinine	0.35 ± 0.02	1.81 ± 0.2*	2.0 ± 0.07*	0.69 ± 0.04*	1.9 ± 0.65*	0.39 ± 0.08

*Note*: GST mRNA isotypes, GST activity, and GST alpha protein levels under control conditions and 18 h following induction of AKI. Significant increases (*) in multiple GST isotypes were observed (*p* < 0.05; outside the control mouse 95% confidence range). Nevertheless, GST activity was significantly depressed (*) in all but the endotoxemic (LPS) model. GST alpha protein levels were increased in each injury model, excepting LPS. Significant azotemia across injury models was observed (BUN, creatinine, mg/dl).

### 3.2 Renal GST isotype mRNA expression under basal conditions and in response to AKI

Although total GST activity was suppressed at 4 h following the induction of glycerol, I/R, and maleate AKI, none of the GST isotypes demonstrated a compensatory mRNA increase (Table 2). Conversely, at 18 h post glycerol, ischemia, and maleate AKI, and at 72 h post cisplatin, 2–10x increases in multiple GST isotype mRNAs were observed (most notably GST alpha and sigma mRNA levels; Table 3), although, as noted above, GST activity remained suppressed. LPS also showed select GST mRNA increases at 18 h post injection, although no associated change in GST activity was observed (Table 3).

### 3.3 Renal GST alpha protein levels

**FIGURE 2:**
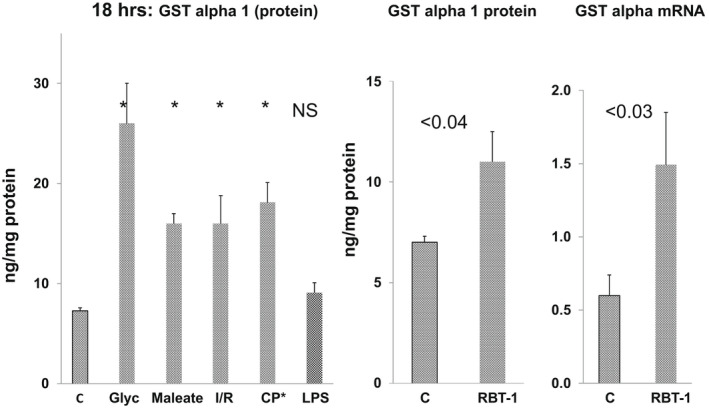
GST alpha 1 protein levels post AKI; GST alpha 1 protein and mRNA levels 18 h post administration of iron sucrose + Sn protoporphyrin (“RBT‐1”). Excepting endotoxemia, each of the AKI models induced a significant increase in GST alpha protein levels (left panel; *p < 0.05) RBT‐1 (Fe sucrose + Sn protoporphyrin) increased GST alpha 1 protein and mRNA levels 18 h post its administration (middle and right panels, respectively). RBT‐1 also significantly increased total GST activity (see Table 5).

Glycerol, I/R, maleate, and cisplatin AKI each induced 2‐4‐fold increases in GST alpha protein levels at 18 h (or 72 h post cisplatin) induction (Figure 2; Table 3). Despite these GST alpha protein increases, total GST enzymatic activity remained suppressed, as noted above.

### 3.4 Urinary total GST activity

At 4 h post maleate injection, an approximate 7x increase in urinary GST activity was observed (controls, 2430 ± 216; maleate 16,261 ± 3925; *p* < 0.025 by unpaired *t*‐test; *p* < 0.001 after log base 10 data conversion).

### 3.5 Ex vivo ischemia‐induced suppression of renal cortical GST activity

When excised kidneys were incubated at 4°C, no significant decline in renal cortical GST activity was observed (0, 2 h, and 4 h activities: 158 ± 10, 160 ± 10, 152 ± 8 (μmol conjugate/ mg issue protein/ min of incubation). Conversely, when incubated at 370 C for 2 and 4 h, progressive GST activity reductions resulted (12% and 20% declines at 2 and 4 h, respectively (158 ± 6, 139 ± 7, 128 ± 5; *p* < 0.005).

### 3.6 Impact of arachidonic acid (C20:4) addition on ex vivo GST activity

Incubation of renal cortical extracts with 50 or 200 μM C20:4 caused prompt, dose‐dependent, reductions in GST activity (135 ± 3; 130 ± 4, 126 ± 3; *p* < 0.005). The 200 μM dose reproduced ~50% of the GST activity suppression that was observed following 4 h of ex vivo whole kidney incubations.

### 3.7 Oxidant preconditioning‐induced GST responses

Oxidant preconditioning with the Fe sucrose/Sn protoporphyrin preparation (“RBT‐1”) failed to evoke a GST mRNA response within 4 h (Table 4). However, by 18 h, a 2.3x GST alpha mRNA increase was observed which corresponded with a 43% increase in GST alpha protein levels (Figure 2, Table 5). The specificity of this GST alpha response was indicated by a lack of an mRNA increase in any of the remaining GST isotypes. Oxidant preconditioning also evoked an approximate 25% increase in total GST activity (Table 5).

**TABLE 4:** GST isotype mRNA levels 4 h after administration of iron sucrose + Sn protoporphyrin “(RBT‐1”)

mRNA	Control	4 h RBT‐1
GST alpha1	0.62 ± 0.08	0.83 ± 0.09 (NS)
GST kappa1	0.34 ± 0.3	0.27 ± 0.01 (NS)
GST mu1	0.66 ± 0.09	0.55 ± 0.04 (NS)
GST mu5	1.72 ± 0.08	1.67. ± 0.09 (NS)
GST omega1	0.39 ± 0.04	0.47 ± 0.05 (NS)
GST pi1	2.06 ± 0.15	2.21 ± 0.23 (NS)
GST sigma1	1.59 ± 0.10	1.31 ± 0.03 (NS)
GST theta1	0.64 ± 0.06	0.55 ± 0.09 (NS)
GST zeta1	1.03 ± 0.03	0.98 ± 0.04 (NS)

*Note*: GST isotype mRNA levels 4 h after administration of iron sucrose + Sn protoporphyrin (“RBT‐1”). No significant changes were observed in any of the assessed mRNAs.

**TABLE 5:** Assessments performed 18 h after administration of iron sucrose + Sn protoporphyrin (“RBT‐1”)

mRNA	Control	18 h RBT‐1
GST alpha1	0.63 ± 0.14	1.49 ± 0.36 (*p* = 0.03)
GST kappa1	0.67 ± 0.06	0.76 ± 0.12 (NS)
GST mu1	0.68 ± 0.11	0.76 ± 0.06 (NS)
GS mu5	1.17 ± 0.11	1.16 ± 0.02 (NS)
GST omega1	0.56 ± 0.08	0.68 ± 0.07 (NS)
GST pi1	1.91 ± 0.08	2.21 ± 0.25 (NS)
GST sigma1	1.59 ± 0.10	1.86 ± 0.40 (NS)
GST theta1	0.64 ± 0.08	0.73 ± 0.14 (NS)
GST zeta1	1.06 ± 0.05	1.03 ± 0.05 (NS)
Total GST activity	148 ± 7	185 ± 10; (*p* = 0.005)
GST alpha 1 (Protein)	7.0 ± 0.3	10.1 ± 1.5 (*p* = 0.04)
BUN	16 ± 2	18 ± 3 (NS)
Creatinine	0.42 ± 0.05	0.45 ± 0.05 (NS)

*Note*: GST isotype mRNA levels, GST alpha protein levels, and total GST activity 18 h after administration of iron sucrose + Sn protoporphyrin (RBT‐1). Significant increase in GST alpha mRNA, GST alpha protein, and total GST activity were observed in the absence of other mRNA responses. BUN and creatinine, mg/dl, remained at normal levels.

## 4 DISCUSSION

The results of these studies demonstrate that acute response to AKI is a rapid (within 4 h) and persistent (≥18 h) decline in renal cortical GST activity. Given that GSTs play an important role in antioxidant defenses, and given that oxidant stress is a critical determinant of AKI, it is plausible that these GST activity reductions contribute to AKI pathogenesis. It is notable that comparable GST activity suppression was observed across the glycerol, maleate, I/R, and cisplatin AKI models, each of which is marked by widespread proximal tubular cell death. Conversely, the LPS/endotoxemia model, which is largely mediated via hemodynamic changes (Mar et al., 2015; Wang et al., 2008; Zager et al., 2006), failed to impact renal GST activity. Thus, these findings serve to link falling GST activity levels with evolving tubular cell death.

To assess GST gene responsiveness in the above AKI models, representative mRNAs for each of the major cytosolic GST classes were assessed. As shown in Table 2, even though GST activity was already suppressed at 4 h post‐injury induction, none of the nine assessed GSTs manifested a compensatory mRNA increase. However, during the AKI maintenance phases, GST alpha, a dominant proximal tubule GST isotype, did mount a strong response, as manifested by 5–10‐fold increases in its mRNA and a 2–3‐fold rise in its protein concentrations. Additionally, GST sigma and theta mRNA increases were observed. Nevertheless, despite these late transcriptional/translational responses, the degrees of GST activity suppression remained at early post‐injury levels. Hence, it appears that the observed GST activity decrements did not reflect failed gene transcription/translation.

Because increased GST urinary excretion is an early marker of AKI (Branten et al., 2000; McMahon et al., 2019; Walshe et al., 2009), we sought to determine whether the observed renal GST activity reductions may have resulted from increased urinary GST excretion. This possibility was suggested by the finding of a 7‐fold increase in urinary GST activity within 4 h of maleate injection. To further explore whether proximal tubule GST excretion is critical to the AKI‐ induced GST activity reductions, we assessed whether ex vivo ischemia could recapitulate this result (i.e., in the absence of urinary flow). These ex vivo ischemia experiments demonstrated approximately 3/4ths the degree of GST activity reductions that were observed following in vivo renal injury. Hence, while urinary GST excretion undoubtedly contributes to the cortical GST activity declines, it cannot, by itself, explain the bulk of the AKI‐ induced cortical GST activity reductions. Similarly, potential GST efflux into plasma as a prime mechanism for renal GST losses was also excluded by these ex vivo ischemia experiments, given the absence of blood flow.

It has been well documented that several cellular lipids, most notably free fatty acids (FFA) and lysophospholipids (LPL), bind to and inhibit GST activity (Boyer et al., 1982; Dixon & Edwards, 2009; Mitra et al., 1991). Given that AKI causes marked free fatty acid and lysophospholipid accumulation (e.g., Alkhunaizi et al., 1996; Portilla, 1999; Venkatachalam et al., 1988; Zager et al., 1991; Zager et al., 1993), we added exogenous arachidonic acid (C20:4) to a renal cortical protein extract and assessed whether a decrease in GST activity resulted. This, indeed, was the case, with the reductions equating to ~50% of those observed during ex vivo kidney ischemia. Clearly, arachidonate addition to renal cortical protein extracts does not recapitulate the accumulation of FFAs and LPLs that occur during AKI. Hence, it seems likely that multiple injury‐induced lipid byproducts act in concert to cause GST activity suppression.

Finally, we considered the possibility that GST activity reductions could arise from increased GST proteolysis. However, two points argue against this possibility. First, GST alpha protein levels markedly rose, rather than fell, in response to glycerol, maleate, I/R, and cisplatin‐induced injury, inconsistent with increased proteolysis. Second, the arachidonate addition experiments were conducted in the presence of protease inhibitors, yet GST activity reductions were still observed. In sum, our results support the notion that GST inhibition, rather than failed gene transcription, GST proteolysis, or urinary/plasma GST losses, is the dominant cause of the observed GST activity declines.

Because oxidative stress is a major determinant of GST gene transcription (Simon et al., 2002), we challenged mice with an oxidant challenge (Fe sucrose/Sn protoporphyrin) to ascertain if GST gene induction would occur. Significant increases in GST alpha mRNA and protein levels were observed. However, none of the other GST isotypes manifested a response. The reason for this GST alpha specificity remains unknown. One possibility is that Fe sucrose and SnPP are preferentially reabsorbed by the proximal tubule (Johnson et al., 2017). This would be expected to preferentially trigger proximal tubule GST expression, and hence, the GST alpha isotype. Of note is that following proximal tubule uptake, Fe sucrose/Sn protoporphyrin induce a cytoprotective state (Johnson et al., 2017; Johnson & Zager, 2018). The present results suggest that GST alpha up‐regulation may play a supporting role in this regard.

Although the present study has provided new mechanistic insights into the fate of GST activity during AKI, we did not address two parallel issues. First, we did not examine which GST isotypes account for the increases in urinary GST activity. Given recent interest in using GST isotypes as AKI markers, in general, and sites of tubule injury, in particular, such an investigation could prove of interest. Second, while it is universally recognized that GST mediates cellular protection via GSH conjugation to free radicals and xenobiotics, we did not confirm that free fatty acid‐induced GST inhibition directly contributes to tubular cell injury (given that this was not the goal of this study). However, it is notable that GST induction has been reported to confer renal protective effects (Pasten et al., 2021), and that provision of the GST substrate, GSH, also mitigates experimental AKI (e.g., ref (Paller, 1998; Sharma et al., 2004). Thus, the literature does imply that fatty acid‐induced GST inhibition could play an injury promoting role. Future studies with graded GST inhibition using either chemical inhibitors or GST knockdown might provide additional confirmatory support for the importance of the GSH/GST antioxidant pathway.

In conclusion, this study documents that a generic response to structural AKI (i.e., evolving lethal proximal tubule injury), is an early and persistent reduction in GST activity. Given the present data, this appears most likely to arise from GST inhibition, rather than from a failure of GST gene transcription, increased proteolysis, or GST losses into urine or plasma efflux. The current results, along with the existing literature (e.g., ref.(Boyer et al., 1982; Dixon & Edwards, 2009; Mitra et al., 1991), imply that injury‐induced generation of GST‐ inhibitory phospholipid degradation products, most notably FFAs and LPLs, are likely candidates in this regard. The role of FFAs and LPLs in evolving AKI has received considerable past attention (e.g., Johnson & Zager, 2018; Portilla, 1999; Zager et al., 1991). The present results add new insight into this investigative area.

## FUNDING INFORMATION

The work was supported by discretionary research funds, Fred Hutchinson Cancer Center, and from a sponsored research agreement, Renibus Therapeutics.

## CONFLICT OF INTEREST

Dr, Zager is a paid consultant to Renibus Therapeutics and Astellas Pharmaceuticals. Ms Johnson is a Renibus employee.

## ETHICS STATEMENT

No human subjects were involved. The animal studies were approved by the Fred Hutchinson Cancer Center IACUC committee.
